# An audit of risk assessments and management for self-harm and suicide in patients with depressive symptoms at a primary care practice in the UK

**DOI:** 10.1192/bjo.2021.271

**Published:** 2021-06-18

**Authors:** Win Let Oo

**Affiliations:** University of Central Lancashire

## Abstract

**Aims:**

Over 5 million adults in England are living with depression, with the highest prevalence rates recorded in the North West and North East of England, 12.88% and 11.53%, respectively (NHS Digital, 2019). Depression is also associated with the highest rates of self-harm and suicide (SH&S) (Singhal, Ross, Seminog, Hawton, & Goldarce, 2014). The impact of SH&S on a family ranges from shock and horror to, blame, secrecy and shame. Survivors may also be negatively judged or self-stigmatise (Cerel, Jordan, & Duberstein, 2008). Managing self-harm episodes has a significant financial implication for the NHS (Tsiachristas, et al., 2017). If high-risk individuals are identified and intervened early, it would not only save lives but also potentially reduce financial strains. The aim of the audit is to evaluate the performance of risk assessment and management of self-harm and suicide at the Reedyford Healthcare Group, Nelson, England, and to determine whether the primary care practice is meeting the standards of the National Institute for Health and Care Excellence (NICE) guidelines for adults with depression.

**Method:**

A retrospective audit of 62 patients presenting with depressive symptoms over 3 months was performed at the Reedyford Healthcare Group.

Two criteria from the NICE guidelines for adults with depression were included with associated standards of 100%:

All patients with depression should be assessed for suicidal ideation and intent by asking direct questions.

A patient presenting with significant risk to self/others should be referred to specialist mental health services the same day, as soon as possible.

**Result:**

42 patients were asked direct questions about SH&S. 2 patients presenting with immediate risk were urgently referred to specialist services. Nonetheless, all those patients at increased risk of suicide were given an increased level of support by the practice. The results indicated that the practice could improve, and a quality improvement approach has been planned.

**Conclusion:**

The assessment of risk in patients presenting with depression is vital. This audit shows that it is not always done in practice. The author has not found other published audits on this topic and suggests that this may be appropriate for a national audit. This is particularly prudent with the current concern regarding mental health in the COVID-19 pandemic.

